# Activation Status of Wnt/ß-Catenin Signaling in Normal and Neoplastic Breast Tissues: Relationship to HER2/neu Expression in Human and Mouse

**DOI:** 10.1371/journal.pone.0033421

**Published:** 2012-03-23

**Authors:** Sara Khalil, Grace A. Tan, Dilip D. Giri, Xi Kathy Zhou, Louise R. Howe

**Affiliations:** 1 Department of Cell and Developmental Biology, Weill Cornell Medical College, New York, New York, United States of America; 2 Department of Pathology, Memorial Sloan-Kettering Cancer Center, New York, New York, United States of America; 3 Division of Biostatistics and Epidemiology, Department of Public Health, Weill Cornell Medical College, New York, New York, United States of America; Roswell Park Cancer Institute, United States of America

## Abstract

Wnt/ß-catenin signaling is strongly implicated in neoplasia, but the role of this pathway in human breast cancer has been controversial. Here, we examined Wnt/ß-catenin pathway activation as a function of breast cancer progression, and tested for a relationship with HER2/neu expression, using a human tissue microarray comprising benign breast tissues, ductal carcinoma *in situ* (DCIS), and invasive carcinomas. Cores were scored for membranous ß-catenin, a key functional component of adherens junctions, and for nucleocytoplasmic ß-catenin, a hallmark of Wnt/ß-catenin pathway activation. Only 82% of benign samples exhibited membrane-associated ß-catenin, indicating a finite frequency of false-negative staining. The frequency of membrane positivity was similar in DCIS samples, but was significantly reduced in carcinomas (45%, *P*<0.001), consistent with loss of adherens junctions during acquisition of invasiveness. Negative membrane status in cancers correlated with higher grade (*P* = 0.04) and estrogen receptor-negative status (*P* = 0.03), both indices of poor prognosis. Unexpectedly, a substantial frequency of nucleocytoplasmic ß-catenin was observed in benign breast tissues (36%), similar to that in carcinomas (35%). Positive-staining basal nuclei observed in benign breast may identify putative stem cells. An increased frequency of nucleocytoplasmic ß-catenin was observed in DCIS tumors (56%), suggesting that pathway activation may be an early event in human breast neoplasia. A correlation was observed between HER2/neu expression and nucleocytoplasmic ß-catenin in node-positive carcinomas (*P* = 0.02). Furthermore, cytoplasmic ß-catenin was detected in HER2/neu-induced mouse mammary tumors. The *Axin2^NLSlacZ^* mouse strain, a previously validated reporter of mammary Wnt/ß-catenin signaling, was utilized to define *in vivo* transcriptional consequences of HER2/neu-induced ß-catenin accumulation. Discrete hyperplastic foci observed in mammary glands from bigenic MMTV/*neu*, *Axin2^NLSlacZ^* mice, highlighted by robust ß-catenin/TCF signaling, likely represent the earliest stage of mammary intraepithelial neoplasia in MMTV/*neu* mice. Our study thus provides provocative evidence for Wnt/ß-catenin signaling as an early, HER2/neu-inducible event in breast neoplasia.

## Introduction

The goal of this study was to investigate the activation status of Wnt/ß-catenin signaling in human breast neoplasia, and to test for a potential relationship between Wnt/ß-catenin pathway activation and expression of human epidermal growth factor receptor 2 (HER2/neu). ß-catenin protein exists in two discrete functional pools in epithelial cells. Membrane-associated ß-catenin is an integral component of the adherens junctions linking membrane-localized E-cadherin to the actin cytoskeleton via alpha-catenin. In contrast, ß-catenin protein accumulates in the cytoplasm and nucleus in response to canonical Wnt signaling, the best characterized pathway regulated by Wnt proteins [1]. Thus the presence of nucleocytoplasmic ß-catenin is considered a hallmark of canonical Wnt pathway activation. Stabilized ß-catenin drives transcriptional activation of multiple protumorigenic genes via interaction with TCF/Lef family transcription factors (http://www.stanford.edu/group/nusselab/cgi-bin/wnt/target_genes). The key role of Wnt/ß-catenin signaling in stem cell biology provides another mechanism by which this signaling axis may impact tumorigenesis [2], [3].

The role of Wnt/ß-catenin signaling in human breast cancer has been subject to much debate [4], [5]. The first mammalian Wnt gene, *Wnt1*, was originally identified as a locus activated by retroviral insertion of mouse mammary tumor virus (MMTV), and transgenic *Wnt1* overexpression was subsequently shown to drive mammary tumor formation in mice [6]–[9]. However, historical failure to identify substantial frequencies of Wnt ligand overexpression in human breast tumors hindered appreciation of the relevance of Wnt signaling to the human disease. Renewed interest followed the identification of ß-catenin/TCF complexes as functional mediators of Wnt-induced transcription.

Striking frequencies of aberrant nucleocytoplasmic ß-catenin accumulation have now been recorded in multiple human neoplastic conditions, most notably in colorectal cancers. Direct elucidation of the likely contribution of Wnt ligand overexpression to pathway activation in human cancer specimens has been hampered by a dearth of immunohistochemistry-compatible anti-Wnt antibodies. However, mutation of pathway components, including the *APC*, *Axin*, and *CTNNB1* genes (encoding ß-catenin), leading to ß-catenin stabilization, and hence activation of the Wnt/ß-catenin pathway, is now recognized as a common event in human tumorigenesis [10], [11]. Because such mutations are comparatively rare in human breast carcinomas, excepting fibromatoses and metaplastic tumors [12]–[21], multiple groups have sought evidence of pathway activation by interrogating the subcellular localization of ß-catenin protein in human breast cancers. Conflicting results from these predominantly immunohistochemical studies have again kindled controversy, with some groups reporting that high proportions of breast cancers have nucleocytoplasmic ß-catenin [22]–[28] but other investigators failing to detect substantial frequencies [29]. Further confusion emanates from several studies which elected not to separately assess the functionally distinct membrane and nucleocytoplasmic ß-catenin pools [30]–[32].

Our goal in the present study was to further investigate the frequency of Wnt/ß-catenin signaling pathway activation in breast neoplasia using ß-catenin immunohistochemistry (IHC) to analyze a human breast tissue microarray (TMA) and to systematically catalogue the prevalence of both membrane-associated and nucleocytoplasmic ß-catenin protein. We sought to characterize the status of Wnt/ß-catenin signaling as a function of breast cancer progression by quantifying and comparing the proportion of tissue samples that exhibited nucleocytoplasmic ß-catenin in benign breast tissue, ductal carcinoma *in situ* tumors, and invasive carcinomas. An additional goal was to assess the relationship between canonical Wnt pathway activation and HER2 overexpression based on several lines of evidence indicating that Wnt and epidermal growth factor receptor (EGFR) signaling pathways can interact [33]–[42]. Data presented herein suggest that Wnt/ß-catenin pathway activation may be an early event in breast neoplasia, and may be driven, at least in part, by HER2/neu expression.

## Materials and Methods

### Ethics Statement

All mice were housed in pathogen-free rooms in filter-topped cages at the Laboratory Animal Research Center at the New York Blood Center. This facility is accredited by the Association for Assessment and Accreditation of Laboratory Animal Care, and operates in accordance with Federal (PHS Policy on the Human Care and Use of Animals, Guide for the Use and Care of Laboratory Animals, Animal Welfare Act), State and local laws and regulations. All mice were used in accordance with protocols approved by the Institutional Animal Care and Use Committees of both the New York Blood Center (Protocol Number 266) and Weill Cornell Medical College (Protocol Numbers 0808-787A, 0055-11). Mice received food and water *ad libitum*.

### Tissue Microarray

To analyze ß-catenin protein immunohistochemically in human breast tissues we used the 2^nd^ Generation Breast Cancer Progression Tissue Microarray developed by the National Cancer Institute (NCI) Cancer Diagnosis Program (CDP). The CDP assembled a collection of 339 breast tissue specimens arrayed on three separate slides (http://cdp.nci.nih.gov/breast/progression_cs2.html). The intended tissue representation was: 69 cores of benign breast tissue (normal or hyperplastic), 31 ductal carcinoma *in situ* (DCIS) specimens, and 239 invasive breast cancers with a principal histology of ductal carcinoma. Of the benign samples, 43 were from women without breast cancer and 26 were from individuals with breast cancer represented elsewhere on the TMA. Of the DCIS samples, 15 were from women without breast cancer and 16 were from individuals with breast cancer represented elsewhere on the TMA. Of the invasive carcinomas, there were 80 from patients that were node-negative at diagnosis, 80 from patients that were node-positive but not metastatic at diagnosis, and 79 from patients with metastatic disease at diagnosis. The TMA slides purchased from the NCI CDP contained coded human biological specimens lacking personal identifiers such that they could not be linked to specific individuals by the research team. Therefore this study did not constitute human subjects research, and Institutional Review Board approval was not required.

Three freshly-cut serial sections were obtained from each of the three TMA blocks. Two of the three sections from each block were stained in duplicate for ß-catenin protein by IHC as described below. The third intermediate section was stained in parallel omitting primary antibody to provide a negative control. Histopathology of each core on slides stained with ß-catenin antibody was evaluated by a breast pathologist (D.D.G.).

### Immunohistochemistry Staining and Scoring

ß-catenin IHC was performed using Clone 14 anti-ß-catenin antibody (BD Transduction Labs) as previously described [43]. Scoring was completed by a specialist breast pathologist (D.D.G.) and a scientist (L.R.H.) blinded to the clinical information; consensus was reached by simultaneous examination using a dual-headed microscope. ß-catenin signal intensity was separately scored in two cellular compartments, membrane and nucleocytoplasmic, which represent distinct functional pools of ß-catenin. The scoring system for each compartment was: 0, no ß-catenin staining; +/−, weak signal; 1+, clear uniform signal; 2+, strong uniform signal; 3+, extremely strong uniform signal. Mean values for each sample were generated from the data for duplicate cores. Membrane and nucleocytoplasmic signals were considered separately when assessing correlations with clinicopathological parameters. For this purpose, cores were assigned as negative (score = 0) or positive (score = +/−, 1+, 2+ or 3+) for each functional pool of ß-catenin.

HER2 IHC was performed on an additional set of TMA slides by the MSKCC Pathology Dept using Ventana's PATHWAY anti-HER2 antibody (clone 4B5), an FDA-approved monoclonal for immunohistochemical detection of HER2 protein in breast cancer tissue. Slides were scored by a breast pathologist (D.D.G.) in accordance with the American Society of Clinical Oncology/College of American Pathologists guideline recommendations for HER2 testing in breast cancer [44].

### Mouse strains, breeding, tissue harvesting and processing

MMTV/NDL mice express a mammary-targeted, mutationally activated *HER2/neu* allele (NDL, Neu Deletion mutant), under the control of the mouse mammary tumor virus (MMTV) long terminal repeat, that induces mammary hyperplasia and tumorigenesis [45]. Tissue sections were prepared from formalin-fixed, paraffin-embedded MMTV/NDL mammary glands (MGs) generated during a previous study [46], and were subjected to ß-catenin IHC as described above.

The *Axin2^NLSlacZ^* strain (*Axin2LacZ*) provides a useful reporter of *in vivo* ß-catenin/TCF signaling. *Axin2* is upregulated in response to canonical Wnt/ß-catenin signaling, and functions as a negative feedback regulator [47], [48]. *Axin2^NLSlacZ^* mice have a bacterial ß-galactosidase (ß-gal; LacZ) expression cassette “knocked-in” to the endogenous *Axin2* locus, such that ß-gal activity provides a surrogate for Wnt/ß-catenin signaling [48]. We have previously established that this strain functions appropriately as a reporter of *in vivo* ß-catenin/TCF pathway activation in mouse mammary gland by demonstrating increased ß-gal activity in response to expression of a *Wnt1* transgene [43].

The MMTV/*neu* strain (FVB/N-Tg(MMTVneu)202Mul/J; The Jackson Laboratory) expresses a wildtype *HER2/neu* allele. MMTV/*neu* females develop palpable mammary tumors with a latency of 2–18 months, with subsequent lung metastases [49]. Mammary tumors in this strain bear mutationally activated alleles of the *HER2/neu* transgene, suggesting that mutational activation of the transgene may be a prerequisite for tumor formation [45], [50].

Here, *Axin2^+/NLSlacZ^* mice were interbred with homozygous MMTV/*neu* animals. Abdominal (#4) mammary glands were harvested post-mortem from both MMTV/*neu* and bigenic MMTV/*neu*, *Axin2^+/NLSlacZ^* female offspring, stained with X-gal to detect ß-gal activity and wholemounted, as previously described [43]. Abdominal MGs from *Pea3^NLSlacZ^* females were harvested and stained in parallel to provide a positive control for LacZ staining. *Pea3^NLSlacZ^* mice (*Pea3LacZ*) have a ß-gal expression cassette “knocked-in” to the endogenous *Pea3* gene, which is expressed predominantly in the myoepithelial compartment in murine mammary gland [43], [51]. Genotyping of *Axin2LacZ* and *Pea3LacZ* mice was performed as described previously [43].

### Data Analysis

In this study, the proportion of samples with positive membrane and nucleocytoplasmic ß-catenin staining in samples of various clinical pathological categories were summarized and compared. Specifically, the associations between ß-catenin staining and the type of tissue sample were examined using mixed-effects logistic regression to take into account possible within-subject correlation because some subjects provided samples of more than one tissue type. Pair-wise comparison of ß-catenin staining positivity between any two tissue types of interest were performed using simultaneous inference methods for general parametric models [52]. P-values were adjusted for multiple comparisons using the conservative Bonferroni method. Fisher's exact test was used to examine the association between ß-catenin staining and clinicopathological parameters within the invasive cancer cases, and to explore the association between membrane and nucleocytoplasmic ß-catenin in samples of the same tissue type.

## Results

In this study, we used IHC to assess ß-catenin protein levels in human breast tissues on the 2^nd^ Generation Breast Cancer Progression TMA generated by the NCI Cancer Diagnosis Program (see Materials and Methods for composition of the TMA set). Meaningful data were obtained from 90% of the 339 cores (Table 1). 19 cores did not contain the stated tissue type, and were excluded from subsequent analyses. A further 15 cores were not assessable because of poor sample integrity. Therefore only 305 cores were further analyzed. Notably, although 97% of the invasive breast cancer cores (231/239) yielded data, only 58% of DCIS cores (18/31) were assessable. Clinical characteristics of the assessed invasive breast cancer cases are summarized in Table 2.

**Table 1:** TMA composition and quality.

Tissue Type	Benign	DCIS	Invasive	ALL
Total no. of cores	69	31	239	339
No. of cores with incorrect tissue	7	7	5	19
No. of unassessable cores	6	6	3	15
No. of data-yielding cores	56	18	231	305
Percent data-yielding cores	81%	58%	97%	90%

**Table 2:** Clinical characteristics of breast cancer patients.

Characteristic	Subcategory	Data
Age at diagnosis, mean±SD		60.5±14.3 years^a^
Race, n (%)	White	200 (87%)
	Black	28 (12%)
	Other	3 (1%)
Grade, n (%)	I	38 (17%)
	II	103 (46%)
	III	83 (37%)
AJCC T stage, n (%)	1	106 (46%)
	2	96 (42%)
	3	10 (4%)
	4	19 (8%)
Metastasis, n (%)		77 (33%)
Tumor size, mean±SD		2.8±1.9 cm
Lymph node involvement, n (%)		110 (58%) b

a Total number of patients = 231.

b Nodal status unavailable for 41 cases with distant metastasis.

**Figure 1:**
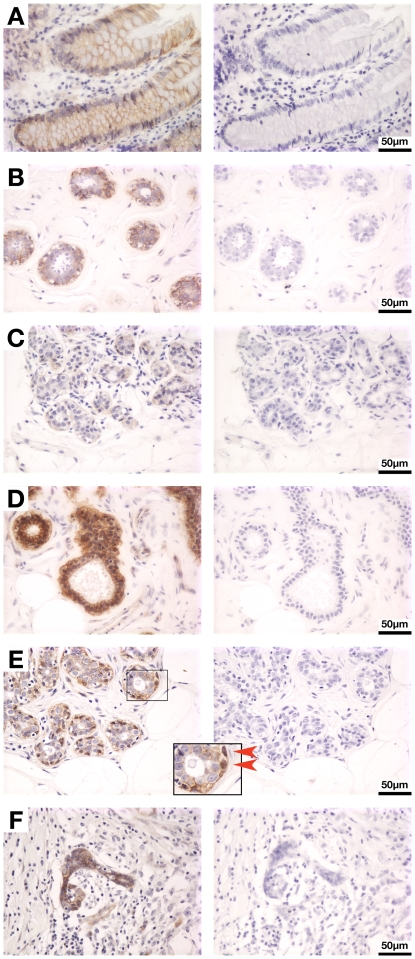
ß-catenin staining patterns of benign human tissues. The 2^nd^ Generation Breast Cancer Progression TMA purchased from the NCI Cancer Diagnosis Program was subjected to ß-catenin IHC using BD Transduction Labs anti-ß-catenin antibody Clone 14 as previously described [43] and counterstained with hematoxylin. As a control, serial sections were stained in parallel omitting primary antibody (right-hand panels). (**A**) Normal colon. (**B–E**) Normal breast. (**F**) Ductal carcinoma *in situ*. The image in (**E**) illustrates the positive nuclear ß-catenin staining observed in myoepithelial cells in some benign breast cores (inset, enlargement of boxed area; red arrowheads indicate positively-staining nuclei).

**Figure 2:**
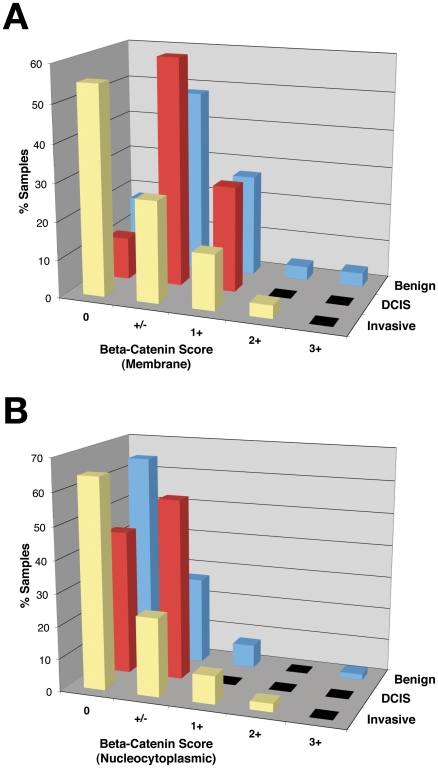
ß-catenin signal as a function of breast cancer progression. Each core was assigned a separate score (0, +/−, 1+, 2+, or 3+) for membrane ß-catenin signal (**A**) and nucleocytoplasmic ß-catenin signal (**B**), and the percentage of cores with each score was separately calculated for benign breast tissue, DCIS, and invasive carcinomas. Of note, the staining pattern denoted as +/− in this study appeared visually similar to the staining pattern scored as 1 by Khramtzov and colleagues [27]. A statistically significant reduction in membrane signal was observed in invasive cancers relative to benign breast tissues (*P*<0.001). A numerical increase in the proportion of DCIS tumors with nucleocytoplasmic ß-catenin compared with normal breast tissue was observed.

We independently scored the signal intensities for two discrete functional pools of ß-catenin, membrane (MB) and nucleocytoplasmic (NC), for each core. Nuclear and cytoplasmic signals were combined into a single entity because accumulation at both these subcellular locations is indicative of canonical Wnt signaling pathway activation. Strikingly in this respect, Kim *et al.* reported an absolute correlation between nuclear and cytosolic ß-catenin protein levels in all of nine breast cancer cell lines tested using a systematic fractionation approach [53]. In our study, ß-catenin signals were detected predominantly in epithelial cells, with negligible staining of stromal components. Normal colon tissue included on the TMA served as a positive control for ß-catenin staining. As expected, colonocytes exhibited distinct membrane staining, with no cytoplasmic or nuclear staining (Figure 1A). We anticipated that normal breast tissues would similarly exhibit membrane-localized ß-catenin, given that ß-catenin is an important junctional component in epithelial tissues. However, only 82% of benign breast samples (46/56) had detectable membrane staining (e.g. Figure 1B), with a mode value of +/− (Figure 2A), and almost one-fifth of benign breast tissues (n = 10) had essentially no detectable membrane signal despite exhibiting overtly normal epithelial morphology (e.g. Figure 1C).

In DCIS tumors the frequency of positive membrane ß-catenin staining was similar to that in benign breast tissue (89% vs. 82%; *P* = 0.72) (Figure 2A; Table 3). In contrast, the proportion of invasive breast cancers with membrane positivity was significantly decreased: only 45% of invasive samples (104/231) exhibited detectable membrane ß-catenin (*P*<0.001 compared with either benign or DCIS). Additionally, when cases were sub-divided into node-negative, node-positive or metastatic at diagnosis, a trend of lower odds of having positive membrane staining in higher stage cancer was observed (Table 3). Examples of invasive cancer staining patterns are shown in Figure S1.

**Table 3:** ß-catenin staining as a function of breast cancer progression.

Stage	MB ß-catenin (pos/total)	MB-positive (%)	*P* a	NC ß-catenin (pos/total)	NC-positive (%)	*P* a
Benign	46/56	82.1%		20/56	35.7%	
DCIS	16/18	88.9%	0.51	10/18	55.6%	0.13
Node-Neg	39/76	51.3%	<0.001	30/76	39.5%	0.67
Node-Pos	32/78	41.0%	<0.001	28/78	35.9%	0.99
Metastatic	33/77	42.9%	<0.001	24/77	31.2%	0.56

a *P*-values were obtained using mixed-effects logistic regression with Benign cases as the reference.

Nucleocytoplasmic staining was not detected in the majority of benign breast samples (Figure 2B). However, approximately one quarter had a +/− nucleocytoplasmic score (15/56), and five samples had stronger staining (e.g. Figure 1D). In total, nucleocytoplasmic staining was detected in 36% (20/56) of benign samples (Table 3; Figure 2B). These findings contrasted with our expectation that benign breast tissue would exhibit little or no nucleocytoplasmic ß-catenin. Similar frequencies of nucleocytoplasmic ß-catenin were observed in invasive carcinomas (82/231; 35%; *P* = 1). Interestingly however, the proportion of DCIS tumors positive for nucleocytoplasmic ß-catenin was quantitatively increased relative to benign tissue (10/18; 56%; Figure 2B; Table 3) (e.g. Figure 1F).

A striking positive correlation was identified between membrane and nucleocytoplasmic ß-catenin positivity in individual samples (Table 4). For carcinoma cores, having positive ß-catenin membrane staining increased the odds of having positive nucleocytoplasmic signal in the same core by 15-fold (95% CI = (7.3, 32.6), *P*<0.001, Fisher's exact test). The two parameters were not associated in DCIS cores, but there was a correlation between membrane and nucleocytoplasmic ß-catenin positivity in benign breast tissue samples (*P* = 0.01, Fisher's exact test). In order to control for effects of this correlation, we conducted an additional analysis restricted to those samples with positive membrane staining (n = 166). In this subset, the proportion of samples with positive nucleocytoplasmic staining was increased for both DCIS (56%, 9/16) and invasive cancer samples (65%, 68/104; *P* = 0.03) relative to benign breast tissue (43%, 20/46).

**Table 4:** Correlations between membrane and nucleocytoplasmic ß-catenin in individual cores.

Tissue Type	MB ß-catenin Status	NC ß-catenin negative/total (%)	NC ß-catenin positive/total (%)	*P* a
Benign	Negative	10/10 (100%)	0/10 (0%)	0.01
	Positive	26/46 (56.5%)	20/46 (43.5%)	
DCIS	Negative	1/2 (50%)	1/2 (50%)	1
	Positive	7/16 (43.8%)	9/16 (56.3%)	
Invasive	Negative	113/127 (89%)	14/127 (11%)	<0.001
	Positive	36/104 (35%)	68/104 (65%)	

a *P*-values were obtained using Fisher's exact test.

Associations between ß-catenin signal and clinicopathological parameters were assessed for the invasive cancer cases (Table 5). Decreased membrane ß-catenin positivity was associated with higher grade (*P* = 0.04) and estrogen receptor (ER) negative status (*P* = 0.03), both indices of poor prognosis. No statistically significant associations were observed between nucleocytoplasmic ß-catenin positivity and individual clinicopathological parameters when all invasive tumors were considered together (Table 5). However, stratification into node-negative, node-positive and metastatic revealed an association between nucleocytoplasmic ß-catenin and HER2/neu positivity in node-positive cases. Nucleocytoplasmic ß-catenin was detected in 67% (8/12) of HER2/neu-expressing node-positive breast carcinomas, but only in 30% (20/66) of HER2/neu-negative node-positive cases (*P* = 0.02). When all invasive cases were considered together, the data were also suggestive of a potential interaction between HER2/neu and ß-catenin stabilization: nucleocytoplasmic ß-catenin was detected in 43.8% of HER2/neu-expressing breast carcinomas, but only in 34.2% of HER2/neu-negative cases (Table 5; not significant). In DCIS tumors, 100% of HER2/neu-positive cases (3/3) exhibited nucleocytoplasmic ß-catenin, whereas only 50% of HER2/neu-negative samples (7/14) were nucleocytoplasmic ß-catenin-positive, but statistical power was limited by small sample size.

**Table 5:** Clinicopathologic association of ß-catenin expression in patients with invasive cancer.

Parameter	Subcategory	MB ß-catenin (pos/total)	MB-positive (%)	*P*	NC ß-catenin (pos/total)	NC-positive (%)	*P*
Age (yr)	<50	25/56	44.6		22/56	39.3	
	≥50	79/175	45.1	1	60/175	34.3	0.52
Tumor size	≤2 cm	52/108	48.1		39/108	36.1	
	>2 cm	52/123	42.3	0.43	43/123	35.0	0.89
Node Status	Negative	38/80	47.5		31/80	38.8	
	Positive	43/110	39.1	0.30	33/110	30.0	0.22
Metastasis	Negative	71/154	46.1		58/154	37.8	
	Positive	33/77	42.9	0.68	24/77	31.2	0.38
Grade	I	16/38	42.1		9/38	23.7	
	II	55/103	53.4		38/103	36.9	
	III	29/83	34.9	0.04^a^	32/83	38.9	0.26
ER	Negative	23/68	33.8		22/68	32.4	
	Positive	80/160	50.0	0.03	59/160	36.9	0.55
PR	Negative	53/118	44.9		46/118	39.0	
	Positive	49/108	45.4	1	35/108	32.4	0.33
HER2	Negative	90/199	45.2		68/199	34.2	
	Positive	14/32	43.8	1	14/32	43.8	0.32

a Overall difference across the three groups. Pairwise comparisons showed significant difference between Grade III cases and Grade II cases (*P*. adj = 0.05, *P* values adjusted for multiple comparisons using Bonferroni method).

**Figure 3:**
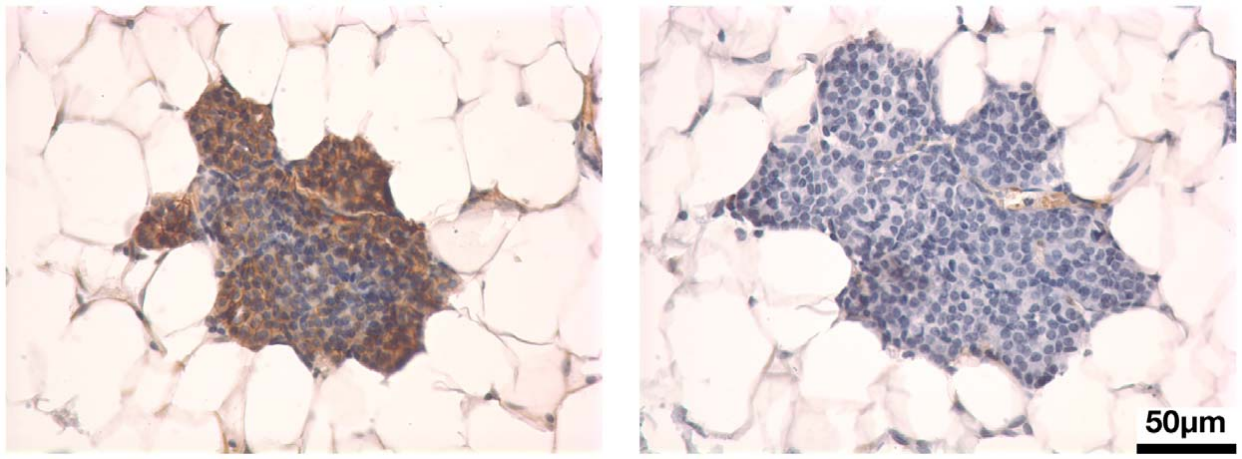
ß-catenin stabilization is evident in mammary precancers in *HER2/neu* transgenic mice. Mammary gland tissue sections from virgin female MMTV/NDL mice were subjected to ß-catenin IHC as previously described [43] and counterstained with hematoxylin. As a control, serial sections were stained in parallel omitting primary antibody (right-hand panel). Diffuse cytoplasmic ß-catenin was detected in DCIS-like lesions in MMTV/NDL MGs.

To further investigate the potential relationship between HER2/neu and canonical Wnt signaling *in vivo* in mammary tissues, we assessed ß-catenin localization and function in mammary glands (MGs) from *HER2/neu* transgenic mice. Firstly, ß-catenin IHC was performed on MG sections from MMTV/NDL mice, which develop multiple DCIS-like lesions in each MG due to expression of a mammary-targeted, mutationally activated *HER2/neu* allele [45], [46]. Diffuse cytoplasmic ß-catenin staining was observed in DCIS lesions in MMTV/NDL MGs (Figure 3), reminiscent of the staining pattern observed in human DCIS tumors (Figure 1F). This staining pattern contrasted with the predominantly membranous signal that we observed in mammary epithelium from wildtype mice [43].

**Figure 4:**
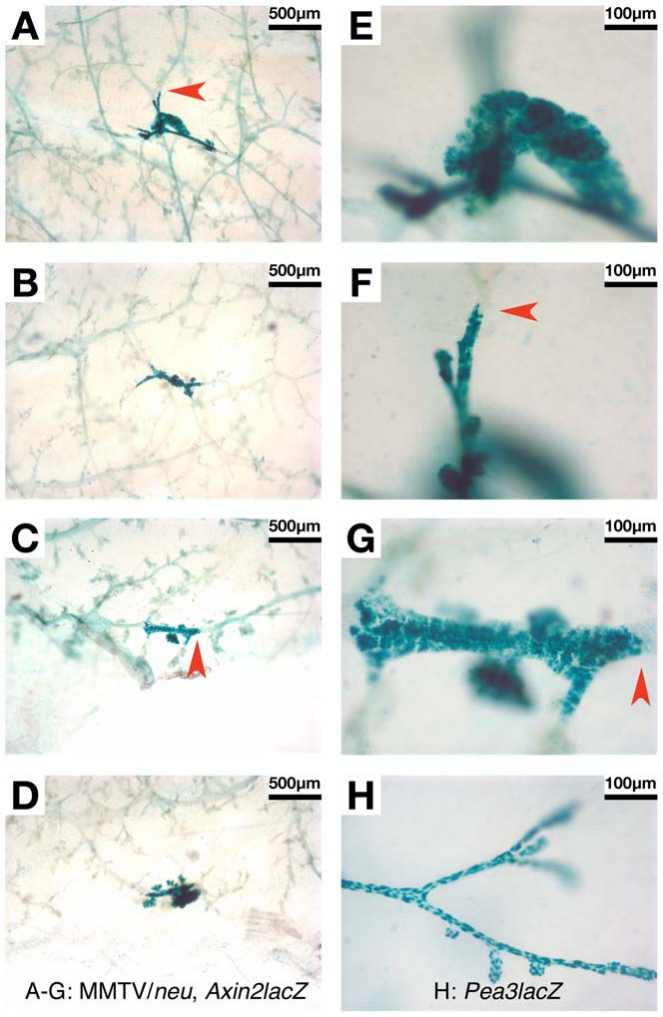
Discrete hyperplastic foci with robust ß-catenin/TCF signaling activity are present in MMTV/*neu* mouse mammary glands. Abdominal (#4) mammary glands were harvested post-mortem from virgin female mice that were MMTV/*neu* or bigenic MMTV/*neu*, *Axin2^+/NLSlacZ^*. MGs were stained with X-gal and wholemounted as previously described [43]. *Pea3^+/NLSlacZ^* (*Pea3LacZ*) samples were processed in parallel for comparison. (**A–D**) Wholemounted glands from bigenic MMTV/*neu*, *Axin2^NLSlacZ^* mice (38–44 weeks old), viewed at 4× magnification. (**E,F**) Higher power images of lesion seen in Panel A, viewed at 20× magnification. (**G**) Higher power image of lesion seen in Panel C, viewed at 20× magnification. (**H**) Wholemounted gland from *Pea3^+/NLSlacZ^* virgin female (39 weeks old) stained in parallel with the specimen in Panels C/G, viewed at 20× magnification. Eight focal lesions displaying intense ß-gal activity, all of similar size, were identified in MGs from 26 bigenic MMTV/*neu*, *Axin2^+/NLSlacZ^* mice. By contrast, no comparable lesions were observed in MGs from MMTV/*neu* mice lacking the *Axin2LacZ* allele (not shown). Additionally, in our previous study of *Axin2LacZ* mice, no such lesions were observed in tissues from *Axin2LacZ* animals lacking a tumor-promoting transgene [43]. Strikingly, each discrete region of robust ß-gal activity in bigenic MMTV/*neu*, *Axin2^NLSlacZ^* MGs coincided with a focus of hyperplastic morphology markedly dissimilar to the normal-looking morphology of the immediately adjacent ductal structures. Also notable were the clearly demarcated boundaries between positive and negatively stained epithelium (examples are marked with red arrowheads in Panels A, C, F, G). The cellular staining profile in bigenic MMTV/*neu*, *Axin2^NLSlacZ^* MGs contrasted with the myoepithelial pattern characteristic of *Pea3LacZ* MGs (compare Panels G and H), suggesting that Axin2LacZ is expressed in luminal cells within the HER2/neu-induced hyperplastic lesions.

To test the *in vivo* functional significance of HER2/neu-induced ß-catenin redistribution, we employed a ß-catenin/TCF reporter strain, *Axin2^NLSlacZ^*, which has a ß-gal expression cassette “knocked-in” to the *Axin2* locus [48]. We previously established the utility of this strain for detecting ß-catenin/TCF-dependent transcription in mouse mammary gland by demonstrating dramatically increased ß-gal reporter activity in *Axin2^NLSlacZ^* MGs in response to expression of *Wnt1*, which activates canonical Wnt signaling [43]. In the present study, we analyzed MGs from bigenic MMTV/*neu*, *Axin2^+/NLSlacZ^* mice, to test the ability of HER2/neu to drive ß-catenin/TCF-dependent transcription *in vivo*. Discrete regions of robust ß-gal activity were detected in the mammary epithelium of bigenic MMTV/*neu*, *Axin2^+/NLSlacZ^* MGs which coincided with focal regions of hyperplasia (Figure 4A–G). Positively staining regions were of similar size in all cases, with clearly demarcated boundaries (e.g. Figure 4A–D, F, G; marked by red arrowheads). The cellular staining profile contrasted markedly with that detected in *Pea3^+/NLSLacZ^* mice (Figure 4H), in which ß-gal expression is predominantly restricted to the myoepithelial layer [43], [51]. Comparison of these staining patterns (e.g. Figure 4G vs 4H) suggests that the Axin2LacZ-positive nuclei in bigenic MMTV/*neu*, *Axin2^+/NLSlacZ^* MGs correspond to luminal epithelial cells, the compartment in which the *neu* transgene is expressed.

## Discussion

In this study we analyzed ß-catenin protein levels in membrane and nucleocytoplasmic compartments in human breast tissues (normal breast, DCIS and invasive cancers) with the goal of better understanding the activation status of Wnt/ß-catenin signaling in human breast cancer, given conflicting findings from previous studies [19], [22]–[32]. Membrane ß-catenin, a key component of adherens junctions, was undetectable in almost one fifth of normal breast tissue cores despite overtly normal epithelial morphology (Figure 2A), suggesting a substantial inherent frequency of false negatives which may reflect loss of ß-catenin antigenicity during tissue processing or storage. Of note, we observed a marked correlation between positivity for membrane and nucleocytoplasmic ß-catenin in individual cores of normal breast tissue (Table 4), again consistent with reduced ß-catenin antigenicity in some samples. This systematic analysis of normal breast tissues stained simultaneously on a TMA highlights the potential for underestimating ß-catenin signal in IHC-based analyses. Nevertheless, we observed a significantly higher frequency of samples lacking discernible membrane ß-catenin among the invasive cores relative to the benign breast samples (55% versus 18%; *P*<0.001 versus benign), consistent with previous reports of reduced membranous ß-catenin in human breast cancers [23], [25], [27], [29], [54], [55]. Loss of adherens junctions has been suggested to contribute to the invasive phenotype [56], [57]. Consistent with this model, there were significant correlations between reduced frequencies of membrane ß-catenin in carcinoma samples and two indices of poor prognosis, higher grade (*P* = 0.04) and ER-negative status (*P* = 0.03).

In light of the fact that nucleocytoplasmic ß-catenin is indicative of activated Wnt/ß-catenin signaling, our analysis of nucleocytoplasmic ß-catenin signal yielded somewhat unexpected data. Specifically, we observed a finite frequency of positive nucleocytoplasmic staining in normal human breast tissues (36%), with 5 of the 56 assessable cores exhibiting substantial signal (Figs. 1D & 2B). These findings contrast with a recent study which did not detect significant nucleocytoplasmic ß-catenin in normal breast [27]. We hypothesized that Wnt/ß-catenin pathway activation observed in benign breast tissue could result from precancerous changes in morphologically normal breast, and further reasoned that breast tissue adjacent to invasive tumors would be more likely to contain such protumorigenic molecular alterations. However, the frequency of nucleocytoplasmic ß-catenin positivity was similar in benign tissue irrespective of the presence or absence of adjacent invasive disease (with invasive disease, 31.6% NC-positive; without invasive disease, 37.8% NC-positive, *P* = 0.77). Thus the basis for ß-catenin stabilization in these samples remains unclear. Notably, the vast majority of previous IHC studies of ß-catenin in breast cancer focused exclusively on malignant specimens.

Intriguingly, some benign breast cores exhibited discrete staining of nuclei in the basal layer of normal-looking epithelium (e.g. Figure 1E, inset, arrowheads). This could reflect pathway activation in these cells by endogenous Wnt ligands, since several *Wnt* genes are expressed in human and mouse breast tissues [58]. Notably, activation of canonical signaling in response to transgenically expressed Wnt1 ligand *in vivo* in mouse MG is predominantly restricted to the myoepithelial compartment [43], [59], reflecting the myoepithelial expression profile of Wnt co-receptors low density lipoprotein receptor-related protein (LRP) 5 and 6 in post-natal MG [60], [61]. The basally restricted expression pattern of LRP5/6 may also provide a partial explanation for the observation that nucleocytoplasmic ß-catenin accumulation is selectively enriched in basal-like breast cancers [19], [27].

We speculate that cells positive for nucleocytoplasmic ß-catenin in the myoepithelial layer may include mammary stem cells, based both on location and on substantial data implicating canonical Wnt signaling in stem cell maintenance [2], [3], [62]. Intriguingly, Zeng and Nusse recently reported evidence for active Wnt/ß-catenin signaling in a subset of basally-located epithelial cells in murine MG which may overlap with the mammary stem cell compartment [63]. Further evidence for a relationship between “stemness” and Wnt signaling is provided by the recently reported association between nuclear/cytoplasmic ß-catenin and the putative stem cell surface marker phenotype CD44^+^/CD24^−^ in human breast carcinomas [27].

In our study, DCIS tumors exhibited the highest frequency of nucleocytoplasmic ß-catenin. Our analysis of Wnt signaling in DCIS was limited by the number of samples that yielded meaningful data (n = 18; Table 1). Nevertheless, we saw a substantially increased frequency of nucleocytoplasmic ß-catenin positivity in DCIS cores relative to that in benign breast samples (56% v. 36%) (Figure 2B, Table 3), suggesting the possibility that Wnt/ß-catenin signaling is activated early during breast neoplasia and could be a target for prophylactic intervention as in colorectal cancer [64].

In invasive carcinomas, we detected nucleocytoplasmic ß-catenin in 35% of samples (Figure 2B), which is within the range detected in other studies [19], [22]–[28], [65]. Notably, and consistent with observations from other investigators [25], [30], [31], [54], we did not see profound levels of nuclear ß-catenin comparable to those reported for colorectal cancers (Figure S1). The intense nuclear signal commonly observed in colorectal tumors may reflect a specific response to *APC* mutation, the causal event in the majority of human colorectal cancers. In contrast, commensurate increases in nuclear and cytoplasmic pools are frequently elicited by other stimuli of the Wnt/ß-catenin pathway, including *Wnt1* transgene expression in mouse MG [43]. Intriguingly, marked cytosolic accumulation of ß-catenin was observed in mitotic cells in some carcinoma samples (Figure S1G, inset), consistent with published data implicating ß-catenin in mitosis [66]–[69].

No correlations were detected between pathway activation and any clinicopathological parameter, when invasive cancers were analyzed as a single population. However, a positive correlation between HER2/neu expression and nucleocytoplasmic ß-catenin was observed in node-positive cases (*P* = 0.02). It is unclear whether this reflects an intrinsic biologic phenomenon specific to the node-positive subset. Nevertheless, the observed association is of particular interest because EGFR family members have the capacity to modulate ß-catenin phosphorylation, localization and transcriptional activity [33]–[40]. In apparent contradiction, other investigators have reported an inverse correlation between HER2/neu expression and nuclear/cytosolic ß-catenin [19], [27]. Conversely, Lopez-Knowles and colleagues identified a relationship between increased cytoplasmic ß-catenin and HER2/neu positivity [28].

To further investigate the relationship between HER2/neu expression and canonical Wnt signaling, we analyzed mammary tissues from *HER2/neu* transgenic mice. Consistent with our observation of an increased frequency of nucleocytoplasmic ß-catenin in human DCIS relative to benign breast epithelium (Figure 2B; Table 3), we detected nucleocytoplasmic ß-catenin in murine mammary DCIS tumors induced by overexpression of a mutationally activated *HER2/neu* transgene (NDL; Figure 3). Functional significance of nucleocytoplasmic ß-catenin in HER2/neu-overexpressing breast neoplasias was suggested by data obtained using the *Axin2LacZ* reporter strain, a validated tool for visualizing ß-catenin/TCF signaling *in vivo* in murine breast tissue. Discrete regions of intense ß-gal reporter activity were observed to colocalize with foci of hyperplastic epithelium in MGs from bigenic MMTV/*neu*, *Axin2LacZ* mice (Figure 4A–G). Notably, all lesions were approximately similar in size and had clearly delineated boundaries, leading us to speculate that these hyperplastic foci with robust ß-catenin/TCF signaling could represent clonal populations with distinct biology from the immediately adjacent ductal epithelium. Importantly in this respect, formation of mammary tumors in the MMTV/*neu* strain is associated with mutational activation of the (initially) wildtype *neu* transgene, which may be an obligate prerequisite to tumor formation [45], [50]. Thus, we speculate that the hyperplastic foci that we observed may be clonal groups of cells bearing a mutated *neu* allele, and potentially the earliest manifestation of mammary intraepithelial neoplasia (MIN) in this strain. Irrespective of the mutational status of the *neu* allele, these observations provide intriguing evidence of ß-catenin/TCF transcriptional activity in *HER2/neu*-expressing MIN lesions, and suggest that HER2/neu-induced activation of canonical Wnt signaling may be an early event in breast neoplasia. Consistent with this model, the elevated frequency at which nucleocytoplasmic ß-catenin was detected in human DCIS specimens compared with invasive carcinomas in our study could reflect the relatively high frequency of HER2/neu overexpression in human DCIS lesions compared with frank cancers [70]–[72].

In summary, in this study we effected a systematic comparison of membrane and nucleocytoplasmic ß-catenin during breast cancer progression from benign breast to metastatic carcinoma. A limitation of the study was that outcome data were not available for the breast cancer cases, and thus prognostic significance of membrane and nucleocytoplasmic ß-catenin positivity could not be determined. However, data addressing prognostic significance has been provided by other studies [19], [23], [27], [28]. Nevertheless, we made several notable findings, including identification of a substantial frequency of canonical Wnt signaling pathway activation in benign human breast tissues, an apparent increase in pathway activation in DCIS tumors, and a correlation between HER2/neu expression and nucleocytoplasmic ß-catenin in node-positive breast carcinomas. A causal relationship between HER2/neu and Wnt/ß-catenin signaling is suggested by our companion mouse studies. We observed ß-catenin accumulation in HER2/neu-induced mouse mammary tumors, and identified discrete regions of ß-catenin/TCF transcriptional activity coincident with early MIN lesions in *HER2/neu* transgenic breast. Our study thus provides provocative evidence for Wnt/ß-catenin signaling as an early, HER2/neu-inducible event in breast neoplasia.

## Supporting Information

Figure S1 **ß-catenin staining patterns of human breast carcinomas.** Images of seven invasive cancer cores demonstrating the range of observed staining patterns. Samples were scored as follows: (**A**) Membrane (MB), 0; Nucleocytoplasmic (NC), 0. (**B**) MB, +/−; NC, 0. (**C**) MB, 1+; NC, 0. (**D**) MB, 1+; NC, 1+. (**E**) MB, 2+; NC, 2+. (**F**) MB, 0; NC, 2+. The image in (**G**) illustrates the positive cytoplasmic ß-catenin staining observed in mitotic cells in some cores (inset, enlargement of boxed area). Left-hand panels; slides treated with primary (anti-ß-catenin) and secondary antibodies; right-hand panels, negative control slides treated with secondary antibody alone.
(TIF)
