# Supplementary figures and images for: Activation Status of Wnt/ß-Catenin Signaling in Normal and Neoplastic Breast Tissues: Relationship to HER2/neu Expression in Human and Mouse
Source: PLoS One. 2012 Mar 23;7(3):e33421. doi: 10.1371/journal.pone.0033421 (PMC3311643; doi:10.1371/journal.pone.0033421)

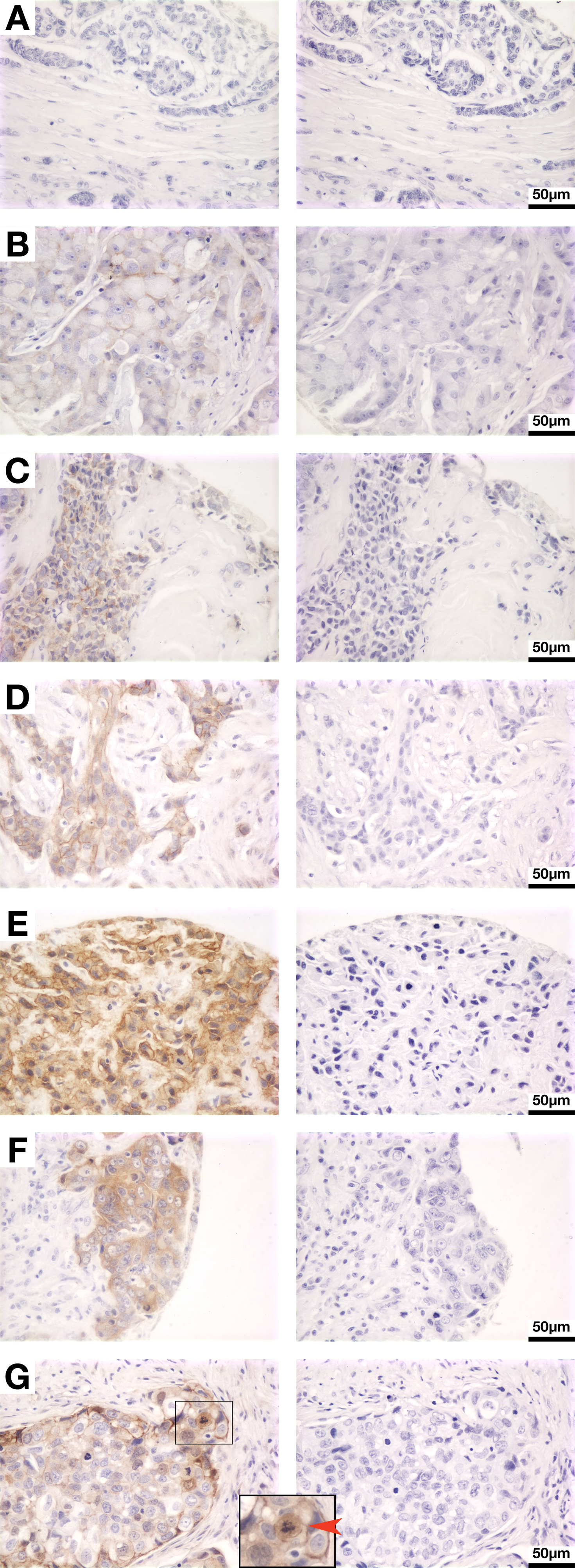

Supplement: Figure S1 — ß-catenin staining patterns of human breast carcinomas. Images of seven invasive cancer cores demonstrating the range of observed staining patterns. Samples were scored as follows: (A) Membrane (MB), 0; Nucleocytoplasmic (NC), 0. (B) MB, +/−; NC, 0. (C) MB, 1+; NC, 0. (D) MB, 1+; NC, 1+. (E) MB, 2+; NC, 2+. (F) MB, 0; NC, 2+. The image in (G) illustrates the positive cytoplasmic ß-catenin staining observed in mitotic cells in some cores (inset, enlargement of boxed area). Left-hand panels; slides treated with primary (anti-ß-catenin) and secondary antibodies; right-hand panels, negative control slides treated with secondary antibody alone. (TIF) [file pone.0033421.s001.tif]
